# Evolution and biological significance of flaviviral elements in the genome of the arboviral vector *Aedes albopictus*

**DOI:** 10.1080/22221751.2019.1657785

**Published:** 2019-08-30

**Authors:** Vincent Houé, Gaelle Gabiane, Catherine Dauga, Marie Suez, Yoann Madec, Laurence Mousson, Michele Marconcini, Pei-Shi Yen, Xavier de Lamballerie, Mariangela Bonizzoni, Anna-Bella Failloux

**Affiliations:** a Department of Virology, Arboviruses and Insect Vectors, Institut Pasteur, Paris, France; b Sorbonne Université, Collège Doctoral, Paris, France; c Institut Pasteur, Center for Bioinformatics, BioStatistics and Integrative Biology (C3BI), Paris, France; d Institut de Biologie Paris-Seine, Paris, France; e Department of Infection and Epidemiology, Institut Pasteur, Epidemiology of Emerging Diseases, Paris, France; f Department of Biology and Biotechnology, University of Pavia, Pavia, Italy; g Aix Marseille Université, IRD French Institute of Research for Development, EHESP French School of Public Health, EPV UMR_D 190 ‘Emergence des Pathologies Virales’, Marseille, France; h IHU Méditerranée Infection, APHM Public Hospitals of Marseille, Marseille, France

**Keywords:** *Aedes albopictus*, arboviral diseases, genetic structure, vector competence, NIRVS

## Abstract

Since its genome details are publically available, the mosquito *Aedes albopictus* has become the central stage of attention for deciphering multiple biological and evolutionary aspects at the root of its success as an invasive species. Its genome of 1,967 Mb harbours an unusual high number of non-retroviral integrated RNA virus sequences (NIRVS). NIRVS are enriched in piRNA clusters and produce piRNAs, suggesting an antiviral effect. Here, we investigated the evolutionary history of NIRVS in geographically distant *Ae. albopictus* populations by comparing genetic variation as derived by neutral microsatellite loci and seven selected NIRVS. We found that the evolution of NIRVS was far to be neutral with variations both in their distribution and sequence polymorphism among *Ae. albopictus* populations. The Flaviviral elements AlbFlavi2 and AlbFlavi36 were more deeply investigated in their association with dissemination rates of dengue virus (DENV) and chikungunya virus (CHIKV) in *Ae. albopictus* at both population and individual levels. Our results show a complex association between NIRVS and DENV/CHIKV opening a new avenue for investigating the functional role of NIRVS as antiviral elements shaping vector competence of mosquitoes to arboviruses.

## Introduction

In less than four decades, the Asian tiger mosquito *Aedes albopictus* (Skuse, 1894) has become a public health concern owing to its ability to transmit several human pathogenic viruses such as Chikungunya virus (CHIKV), Dengue viruses (DENV) and Zika virus (ZIKV) [1]. This vector is responsible of several recent arboviral outbreaks (reviewed by [2]) and astonished by the speed at which it has conquered all continents except Antarctica [3,4], thus becoming one of the world’s 100-most invasive species according to the Global Invasive Species Database. Originally native to tropical forests of South-East Asia, *Ae. albopictus* was confined for centuries to few regions in Asia. Starting in the eighteenth or nineteenth centuries, *Ae. albopictus* was introduced in the islands of the Indian Ocean by Asian immigrants. From the late 1970s, this species took advantage of the increase in global trade to invade most tropical and sub-tropical regions of the world [5]. Additionally, the capacity of its eggs to undergo photoperiodic diapause in winter, favoured *Ae. albopictus* colonization into temperate regions [6]. *Aedes albopictus* was first reported in Europe in 1979 [7], in North America in 1985 [8], in South America in 1986 [9] and in Africa in 1990 [10].

Migratory routes used by *Ae. albopictus* to expand from its Asian cradle can be defined using population genetic approaches [11–13]. Mosquito populations form groups of interbreeding individuals, which coexist in space and time. These genetic units are interconnected through gene flow. The current worldwide distribution of *Ae. albopictus* is a direct consequence of increasing human activities with rapid irradiation of populations [14–18]. Populations established locally presented a high genetic variability evidencing a mixture of individuals of distinct origins with disparate susceptibilities to arboviruses, which are reflected by different vector competences [17]. Vector competence refers to the ability of a mosquito population to become infected after an infectious blood meal, to support viral replication, dissemination and transmission to a new host in a subsequent blood-meal [19]. The level of vector competence depends on the tripartite interactions among the mosquito genotype, the virus genotype and environmental factors under GxGxE interactions [20].

In the early 2000s, non-retroviral integrated RNA virus sequences (NIRVS) were discovered in different metazoans, including mosquitoes [21,22]. *Aedes* mosquitoes can host NIRVS originated from different viruses related to arboviruses: mainly insect-specific viruses (ISVs) including insect-specific flaviviruses (ISFs) and other viruses belonging to the *Mononegavirales* order (such as rhabdoviruses) [22–25]. Additionally, most NIRVS correspond to fragmented viral open-reading frames, are flanked by transposable elements (TEs), enriched in PIWI-interacting RNA (piRNA) clusters and produce piRNAs [29]. piRNA clusters are genomic regions composed of fragmented sequences of TEs which are expressed as long primary single-stranded RNAs and processed into fragments of 24–30 nucleotides called piRNAs. In the model organism *Drosophila melanogaster*, piRNAs are primarily produced in germline cells and target TE transcripts based on sequence complementarity to protect from heritable lesions [26–29]. The landscape of TE fragments within piRNA clusters defines the regulatory properties of *D. melanogaster* strains to TE invasion [30]. The analogy between TE fragments and viral sequences in piRNA supports the hypothesis that viral sequences may contribute to mosquito susceptibility to subsequent viral infections. If viral integrations contribute in controlling virus replication, with consequences on vector competence, positive selection should be expected [31]. On the contrary, if NIRVS stand for fossil records, these sequences should reach fixation and evolve at a neutral rate [32,33].

In this study, we selected 19 *Ae. albopictus* populations that cover the geographical distribution of the species, where CHIKV and DENV were circulating, to study the evolutionary dynamics of seven selected NIRVS [24]. The occurrence of NIRVS in populations was compared to processes driving population genetic differentiation observed on neutral loci (i.e. microsatellites). We showed that (i) based on microsatellite marker polymorphism, populations of *Ae. albopictus* are distributed into two different genetic clusters, one of them divided into four subclusters, without any correlation between genetic and geographical distances, (ii) the distribution of NIRVS in geographic populations and their polymorphism are not related to genetic divergences within and between populations as depicted by microsatellite markers, suggesting that NIRVS are not evolving neutrally, and (iii) all NIRVS studied, except AlbFlavi1 may have influence on vector competence to DENV and CHIKV, at the population level.

## Materials and methods

### Ethic statements

Mice were housed in the Institut Pasteur animal facilities accredited by the French Ministry of Agriculture for performing experiments on live rodents. Work on animals was performed in compliance with French and European regulations on care and protection of laboratory animals (EC Directive 2010/63, French Law 2013-118, February 6th, 2013). All experiments were approved by the Ethics Committee #89 and registered under the reference APAFIS#6573-201606l412077987 v2.

### Mosquitoes

To assess genetic variation within and among *Ae. albopictus* populations, 19 samples from different geographic locations were studied (Table 1). These populations ranging from 10 to 30 mosquitoes were selected in the native range of *Ae. albopictus* where arboviral outbreaks and epidemics occurred [17,34,35]. Only the 13 populations of 19–20 mosquitoes and the Foshan colony used as control were selected to characterize NIRVS polymorphism [36].

**Table 1.** Details on *Aedes albopictus* populations analyzed.

	Population name	Continent	Country	City	Generation	Year of collection	Mosquitoes	
							females	Males
1	Alessandria	Europe	Italy	Alessandria	F2	2012	10	10
2	Ulcinj	Europe	Montenegro	Ulcinj	F1	2013	10	10
3	Cagnes-sur-mer	Europe	France	Cagnes-sur-mer	F13	2000	10	0
4	Montsecret	Europe	France	Montsecret	F4	2002	0	10
5	Bar-sur-Loup	Europe	France	Bar-sur-Loup	F1	2011	10	0
6	Tirana	Europe	Albania	Tirana	F6	2016	15	15
7	Franceville	Africa	Gabon	Franceville	F2	2015	10	10
8	Mfilou	Africa	Congo	Mfilou (Brazzaville)	F3	2012	10	10
9	Bertoua	Africa	Cameroon	Bertoua	F5	2008	10	4
10	Saint-Denis	Africa	La Réunion	Saint-Denis	F2	1998/2006	10	10
11	Rabat	Africa	Morocco	Rabat	F1	2017	15	15
12	Vero Beach	America	USA	VeroBeach	F5	2016	10	10
13	Rio	America	Brazil	Rio de Janeiro	F1	2001	10	9
14	Jurujuba	America	Brazil	Jurujuba	F1	2014	10	0
15	Manaus	America	Brazil	Manaus	F1	2015	15	15
16	PMNI	America	Brazil	PMNI (Nova Iguaçu)	F1	2015	15	15
17	Binh Duong	Asia	Vietnam	Binh Duong (Ben Cat) Phu Hoa	F9	2014	10	10
18	Sarba	Asia	Lebanon	Sarba	F0	2011	10	10
19	Foshan	Asia	China	Foshan	Lab colony	–	10	10
20	Oahu	America	Hawaii	Oahu	Lab colony	1999	10	10

Note: Except the colony Foshan, all 19 populations were genetically characterized using 10 microsatellite markers, and 13 populations (in bold) were selected for studying NIRVS diversity.

Mosquitoes were collected in the field as immature stages (larvae, pupae, eggs). Frozen mosquitoes (<13th generation) were used for population genetic and NIRVS analyses. For the pilot experiments for DENV and CHIKV infections, F1 mosquitoes from Tibati population (Cameroon) and F18 mosquitoes from Foshan laboratory strain were used. Larvae obtained after immersion in dechlorinated tap water of field-collected eggs were distributed in pans of 200 individuals. Immature stages were fed every two days with a yeast tablet dissolved in 1 L of dechlorinated tap water and incubated at 26 ± 1°C. Emerging adults were placed in cages and maintained at 28 ± 1°C with a light/dark cycle of 12/12 h at 80% relative humidity and supplied with a 10% sucrose solution. Females were exposed three times a week to anesthetized mice (OF1 mice; Charles River Laboratories, MA, USA) as a source of blood for producing eggs.

### Microsatellite genotyping

Genomic DNA was extracted from individual mosquito using the Nucleospin Tissue kit (Macherey-Nagel, Hoerdt, France) according to manufacturer’s instructions. Briefly, mosquitoes were individually homogenized in 180 µL lysis buffer supplemented with 25 µL of Proteinase K. To bind total nucleic acids, homogenates were passed through columns. Silica membranes were further desalted and DNA was collected in 100 µL of elution buffer. Quality and quantity of DNA were then assessed using the Nanodrop 2000 Spectrophotometer (*Thermo* Scientific™, MA, USA) and a PCR was performed using *histone h3* reference gene (NCBI: XM_019696438.1) as control. Eleven microsatellite loci were amplified using PCR specific primers flanking the repeated region [16]. PCR reaction mixtures in a final volume of 15 µL contained 50 ng genomic DNA, 1X PCR buffer, 1.5 mM MgCl2, 0.27 mM dNTPs (Invitrogen™, CA, USA), 1U Taq polymerase (Invitrogen™), and 10 µM of each primer (one was 5′ labelled with a fluorescent dye). PCR cycling conditions consisted in a step at 94°C for 5 min, followed by 29 cycles at 94°C for 30 s, 58°C for 30 s, and 72°C for 30 s with a final step at 72°C for 10 min. Aliquots of PCR products were visualized in 2% agarose gels stained with ethidium bromide under UV light. Each PCR product was then diluted 1:10 in ddH2O water and 2 µL of this dilution was added to 10 µL of a mixture of deionized formamide and GeneScan-500 ROX size standard (Applied Biosystems, CA, USA). Genotyping was processed in an ABI3730XL sequence analyser (Applied Biosystems) and data analyzed using GeneScan and Genemapper softwares.

### Genetic diversity of populations

For statistical analyses, mosquitoes collected from each sampling site were assumed to represent local populations.

Amounts of heterozygosity at various levels of population structure were explored by using *F*_IS_ and *F*_ST_ values. The *F*_IS_ inbreeding coefficient indicates the level of heterozygosity within each population. The *F*_ST_ fixation index measures the reduction in heterozygosis due to random genetic drift between populations. The Wright’s *F*-statistics were computed in Genetix v.4.05.2. software [37] and tested using 10^4^ iterations according to Weir and Cockerham (1984). The number of alleles (*N_A_*), allelic richness (*A*_R_), expected (*H*_E_), observed (*H*_O_) heterozygosis, *F*_IS_ by locus and *F*_ST_ by populations were obtained. The significance of *F*_IS_ and *F*_ST_ were analyzed using FSTAT v.2.9.3.2 [38].

Departures from Hardy-Weinberg equilibrium, linkage disequilibrium between loci and molecular variance (AMOVA) over all populations were analyzed with Arlequin v3.5.2.2 to estimate intra- and inter-population variation [39]. The mean frequency of null alleles (a mutation in microsatellite flanking regions leading to an absence of amplification products) per population was also very low (i.e. 0.06) and ranged from 0.000 to 0.118, meaning that the selected microsatellite loci were successfully amplified and appropriate for population genetic analysis. Relationships between geographical and genetic *F*_ST_ distances were tested between populations using Mantel test implemented in GenAlEx v 6.5 [40].

### Graphic representation of relatedness among populations

Genetic relationships between populations were estimated by using PHYLIP 3.69, as previously described [41]. Cavalli-Sforza & Edwards’s (CSE) chord distance for each pair of populations was calculated (GENDIST module). The resulting distance matrix was used to create a phylogram based on Neighbour-Joining (NEIGHBOUR module). Node confidence was inferred via 100 bootstrap replicates (modules SEQ- BOOT, GENDIST, NEIGHBOUR and CONSENSE).

### Test of isolation by distance

To test the hypothesis whether the geographical pattern of genetic differentiation is caused by isolation by distance (IBD), we ran Mantel tests for pairwise matrices between geographical distances (kilometres) and genetic differentiation (measured as *F*_ST_/(1−*F*_ST_)).

### Genetic structure of populations

Genetic population structure was assessed with individual-based Bayesian clustering method implemented in the program STRUCTURE v.2.3.4 [42]. The likelihood of each possible number of genetic populations (K), ranging from 1 to 20, was calculated after 10 independent runs for each value of K, using a burn-in of 500,000 replications, 500,000 Markov Chain Monte Carlo steps, assuming an admixture model, with frequencies correlated between populations and without the use of sampling location as a prior. The most likely number of populations (K) was estimated by the ΔK method described by Evanno et al. [43] with Structure Harvester software (http://taylor0.biology.ucla.edu/structureHarvester/). The results were then graphically displayed with DISTRUCT 1.1 [44].

### NIRVS in natural populations

Six NIRVS (AlbFlavi1, AlbFlavi2, AlbFlavi4, AlbFlavi10, AlbFlavi36, AlbFlavi41), plus CSA [21], were studied. They were chosen based on their unique occurrence in different regions of the Foshan genome, except for AlbFlavi41, known to be duplicated. Because of its length [21], CSA was characterized using two sets of primers (CSA-NS3 and CSA-JJL), and considered in analysis as two separated datasets (Supplementary Table 1). NIRVS were searched on the same mosquitoes as those used for microsatellite genotyping.

PCR primers flanking each NIRVS (Supplementary Table 1) were designed using PRIMER3 [45]. PCR reactions were performed in a final volume of 25 μL using 5 ng of DNA, PCR buffer 1X, 2.9 mM MgCl_2_, 0.25 mM dNTP mix, 0.25 μM of each primer and 1.25 unit of *Taq* DNA polymerase (Invitrogen™). Amplifications were done using *T100*™ *Thermal Cycler* (Bio-Rad) according to the following cycle conditions: 95°C for 5 min, 35 cycles at 95°C for 15 s, 59–64°C for 90 s, 72°C for 90 s, and a final elongation step at 72°C for 10 min. PCR products were electrophoresed on 1.5% of agarose gels stained with ethidium bromide and visualized under UV light. All negative samples were confirmed with a second-round PCR.

The number of each NIRVS per population (frequency) was represented using Graph Pad Prism version 6 (Graph Pad Software, CA, USA).

### NIRVS between populations

NIRVS composition of each population was expressed in terms of relative abundance corresponding to the percentage of each NIRVS relative to the total number of tested mosquitoes. To assess variations of NIRVS abundance between populations, we calculated Bray Curtis dissimilarities, a metrics, widely used, including data standardization [46]. A dendrogram was generated by using this dissimilarity matrix and neighbour-joining method, to visualize NIRVS compositional differences across populations [46].

### Mantel tests between NIRVS and microsatellites

Mantel tests implemented in GenAlEx v 6.5 [40], were used to evaluate the statistical significance of the correlation between two or more distance matrices, using permutation tests. The significance of associations between matrixes of $\phi^{\prime }st$ distance among populations as derived by microsatellite markers or NIRVS distribution was tested using the Mantel test with 999 permutations.

### NIRVS association with vector competence of *Ae. albopictus* at the population level

Vector competence is assessed using several parameters [47]. The dissemination efficiency (DE) refers to the proportion of mosquitoes able to disseminate the virus beyond the midgut barrier after ingestion of infectious blood meal and active viral replication in midgut epithelial cells. Published data on DEs to DENV and CHIKV were retrieved for mosquito populations with close or identical geographical proximity with the populations analyzed (Supplementary Table 2). DEs were described using median and inter-quartile range (IQR). Logistic linear regression models were used to test association between the presence of NIRVS and DEs at the *Ae. albopictus* population level. *P*-values <0.05 were considered significant.

### Sequence polymorphism and evolutionary dynamics of AlbFlavi2 and AlbFlavi36

#### Sequence polymorphism of AlbFlavi2 and AlbFlavi36

Amplification products for AlbFlavi2 and AlbFlavi36 were purified by using NucleoSpin® Gel and PCR Clean-up kit (Macherey-Nagel) according to the manufacturer’s instructions and sequenced by Eurofins Genomics (Cochin hospital platform, Paris, France). If the quality of chromatogram profiles assessed with Geneious® 10.1.3 did not meet the standard required, amplicons were subsequently cloned into pCR™II-TOPO® vector using *TOPO*® *TA Cloning*®*Kit (Thermo Fisher Scientific) and transformed into One Shot*® *TOP10* Chemically Competent *E. coli* (Invitrogen™). Sequences were aligned in Geneious® 10.1.3 using MUSCLE algorithm [48]. p-distance values, representing proportion of nucleotide sites at which two sequences being compared are different, were calculated after alignments using MEGA 10.0.5.

#### Phylogeny based on AlbFlavi2 and AlbFlavi36

DNA sequences from AlbFlavi2 and AlbFlavi36 were aligned with MUSCLE algorithm [48]. Exogenous virus sequences were used as outgroup to determine the direction of character transformations. Phylogenetic trees were obtained by parsimony analysis implemented in the PAUP* software package (version 4.0), by using gap as 5th state or not (data not shown) and nearest neighbour interchange (NNI) for tree rearrangements [49].

### Pilot experiment assessing the association between AlbFlavi2/Albflavi36 and vector competence of *Ae. albopictus* at the individual level

Logistic linear regression models were used to test association between the presence of AlbFlavi2 and AlbFlavi36 and viral dissemination in single mosquitoes from the Foshan colony and a field-collected African population (Tibati collected in Tibati, Cameroon in 2018). Foshan and Tibati mosquitoes were experimentally infected with DENV (DENV-2, Accession number: MK268692 [50]) and CHIKV (06.21, Accession number: AM258992 [51]) provided in blood meals as described in Amraoui et al. 2019 [52]. All surviving mosquitoes were examined individually at 14 days post-infection to define (i) the infectious status; presence of infectious particles in heads was estimated by focus fluorescent assay on C6/36 *Ae. albopictus* cells [52], and (ii) the presence of AlbFlavi2 and/or AlbFlavi36 by PCR on DNA extracted from bodies and thorax. Statistical analyses were conducted using the Stata software (StataCorp LP, Texas, and USA). *P*-values <0.05 were considered significant.

## Results

### Characterization of populations using microsatellite data

A total of 363 individuals (Table 1) were genotyped at 11 microsatellite markers. Ten of the 11 microsatellite loci, by showing a frequency upper to 0.95 for the most common allele, were considered polymorphic and as such, were included in the analysis. Only the A17 locus was monomorphic and then excluded from the analyses. No significant linkage disequilibrium between any pairs of loci was observed indicating that the 10 loci were statistically independent from each other. Each locus was tested for within-population deviations from HWE implementing Bonferroni correction for multiple testing: 20 deviations out of 190 combinations were found but did not cluster at any population or locus.

The loci displayed a mean PIC of 0.59 suggesting that they were sufficiently informative for assessing the degree of variability and structuring of mosquito populations (Table 2). The number of alleles, averaged over all loci, ranged from 4 to 19, with a mean value of 10.9 per locus, and a mean allelic richness at 2.44 (Table 2). Eight of the 10 loci presented a significant difference between observed and expected heterozygosity, with a mean *F*_IS_ of 0.17 (*p*-value <0.001; Table 2), supporting an excess of homozygotes in *Ae. albopictus* populations.

**Table 2.** Genetic diversity at each microsatellite locus for all mosquito populations.

	*N_A_*	*N*	Allelic richness	PIC	*H*_o_	*H*_E_	*F*_IS_
A1	4	288		0.44	0.16	0.47	0.59
A2	10	360	2.43	0.55	0.63	0.62	−0.15
A3	18	341	3.14	0.74	0.62	0.78	0.08
A5	19	334	2.90	0.75	0.52	0.78	0.13
A6	11	330	2.59	0.68	0.54	0.68	−0.04
A9	9	326	2.73	0.65	0.59	0.70	0.01
A11	9	323	2.81	0.71	0.53	0.75	0.15
A14	9	351	1.71	0.41	0.17	0.40	0.38
A15	6	347	1.25	0.13	0.04	0.15	0.57
A16	14	279		0.83	0.66	0.84	0.04
Mean	10.9	327.9	2.44	0.59	0.45	0.62	0.17

Notes: *N_a_*, number of alleles; *N*, number of individuals examined at a locus; PIC, polymorphism information content; *H*o, observed heterozygosity; *H*e, expected heterozygosity; *F*_IS_, the inbreeding coefficient. Values in bold are significant at the 0.1% after Bonferroni correction.

#### Population diversity

The overall mean number of alleles per population was 3.7 varying between 2.0 and 5.0. Private alleles were rarely found, their mean frequency per population ranged from 0.00 to 0.079 (Table 3). Mean *F*_IS_ value per population was −0.09, with values ranging from −0.0285 to 0.338 (Table 3). Estimation of the molecular variance within and among populations (AMOVA) revealed that most of the variation (89.6%) was detected within individuals whereas only 10.4% occurred among populations.

**Table 3.** Analysis of genetic variability of different geographical populations of *Aedes albopictus.*

Populations	Country	Continent	*N*	*n_a_*	*n_a_*/*n*	*n_p_*	*n_p_*/*n*	*A_p_*	*H*o	*H*_E_	*A_n_*	*F*_IS_
Vero Beach	USA	America	20	4.4	0.22	2	0.10	0.07	00.48	0.53	0.08	0.12***
Oahu	Hawaii		20	2.6	0.13	0	0.00	0.00	0.25	0.34	0.08	0.30***
Manaus	Brazil		30	3.4	0.11	3	0.10	0.07	0.32	0.47	0.12	0.34***
PMNI			30	3.6	0.12	1	0.03	0.03	0.39	0.51	0.11	0.25***
Rio			19	3.4	0.18	0	0.00	0.00	0.42	0.50	0.08	0.18***
Jurujuba			10	3.3	0.33	0	0.00	0.00	0.47	0.42	0.00	−0.06
Montsecret	France	Europe	10	2.0	0.20	0	0.00	0.00	0.50	0.37	0.01	−0.29
Cagnes-sur-Mer			10	2.8	0.28	1	0.10	0.06	0.55	0.46	0.01	−0.14
Bar-sur-Loup			10	3.8	0.38	2	0.20	0.08	0.50	0.52	0.05	0.13*
Alessandria	Italy		20	4.1	0.21	2	0.10	0.05	0.43	0.50	0.06	0.18***
Ulcinj	Montenegro		20	3.1	0.16	0	0.00	0.00	0.40	0.39	0.03	0.02
Tirana	Albania		30	3.8	0.13	3	0.10	0.07	0.35	0.43	0.08	0.21***
Rabat	Morocco		30	3.2	0.11	0	0.00	0.00	0.42	0.47	0.06	0.11***
Bertoua	Cameroon		14	4.3	0.31	1	0.07	0.07	0.46	0.52	0.08	0.16***
Franceville	Gabon	Africa	20	4.8	0.24	1	0.05	0.03	0.44	0.56	0.08	0.25***
Mfilou	Congo		20	4.0	0.20	0	0.00	0.00	0.60	0.55	0.02	−0.06
Saint-Denis	La Réunion		20	5.1	0.26	6	0.30	0.05	0.52	0.55	0.07	0.08
Sarba	Lebanon	Asia	10	3.5	0.35	4	0.40	0.08	0.55	0.44	0.00	−0.18
Binh Duong	Vietnam		20	5.0	0.25	3	0.15	0.08	0.2	0.41	0.05	0.11**
Mean			19.1	3.7	0022	1.5	0.09	0.04	0.45	0,47	0.06	−0.09

Notes: *N*, population size; *n_a_*, mean number of alleles; *n_a_*/*N*, mean number of alleles/individual; *n_p_*, number of private alleles; *n_p_*/*N*, mean number of private alleles/individual; *A_p_*, mean frequency of private alleles; *H*_O_, mean observed heterozygosity; *H*_E_, mean expected heterozygosity; *A*_n_, mean frequency of null alleles; *F*_IS_, the inbreeding coefficient.

**p-value ≤ 0.01; ***p-value ≤ 0.001.

#### Population genetic structure

**Figure 1.**
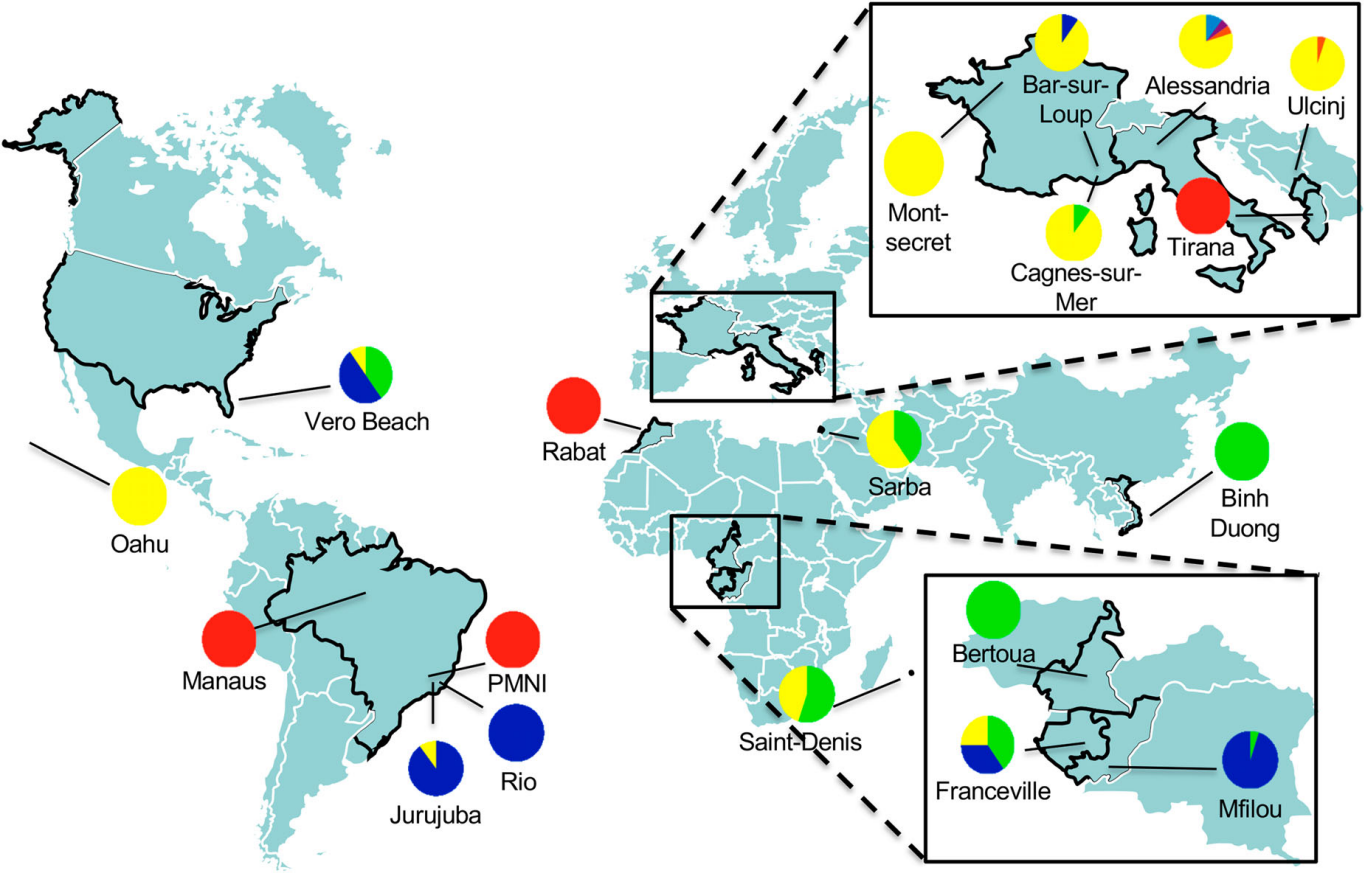
Estimated population structure of 363 individuals (19 populations) using 10 microsatellite markers. Map with sampling sites of populations with colour pie charts showing genotype frequencies, according to Cluster 1 (red) and Cluster 2, which the latest subdivided into 4 subclusters (blue, green, yellow and orange), deduced from the ΔK curve obtained (Supplementary Figure 1(A–C)).

The overall differentiation across all 19 populations was high (*F*_ST_ = 0.239) with *F*_ST_ being highly significant (*p*-value <0.001), suggesting a significant genetic structure. To identify genetic clusters among tested individuals, we implemented 25 independent simulations in the software Structure according to the method of Evanno; the uppermost level of structuring in the model was observed at K = 2 (ΔK = 2293.4; Supplementary Figure 1(A)). The best assignment of individuals made by Structure led to two clusters: (i) cluster 1, which includes populations from Brazil (i.e. PMNI and Manaus), Northern Africa (i.e. Rabat) and a population from Albania, and (ii) cluster 2, which includes the remaining populations (Figure 1 and Supplementary Figure 1(B)). Although Structure identified only two clusters, their genetic differentiation was significantly high (*F*_ST_ = 0.12, *p*-value ≤ 0.001). Additionally, these two genetic clusters were found to deviate significantly (*p*-value ≤ 0.01) from Hardy-Weinberg proportions, with *F*_IS_ values of 0.23 and 0.36, respectively after implementing 10^4^ permutations on allele frequencies in the software GENETIX. This result suggested a Wahlund effect which indicated a genetic substructure. As a consequence, we re-analyzed the two clusters separately and detected substructures in Cluster 2 (Figure 1 and Supplementary Figure 1(C)). Sub-structuring of Cluster 2 emphasized differentiation among populations from South America (Jurujuba and Rio), Europe (i.e. all French and Italian populations), Africa (Bertoua and Mfilou) and Asia. On the contrary, populations from Florida (Vero Beach), Middle East (Sarba), La Reunion Island (Saint Denis) and Gabon (Franceville) appeared genetically mixed suggesting that these sites may represent cross-roads in *Ae. albopictus* colonization out of Asia (Figure 1).

#### Spatial and genetic data

When considering the genetic differentiation according to geographical distances using Mantel test, only 21% of the genetic variability was explained by the geographical distance (*p*-value = 0.03).

### NIRVS analysis

**Figure 2.**
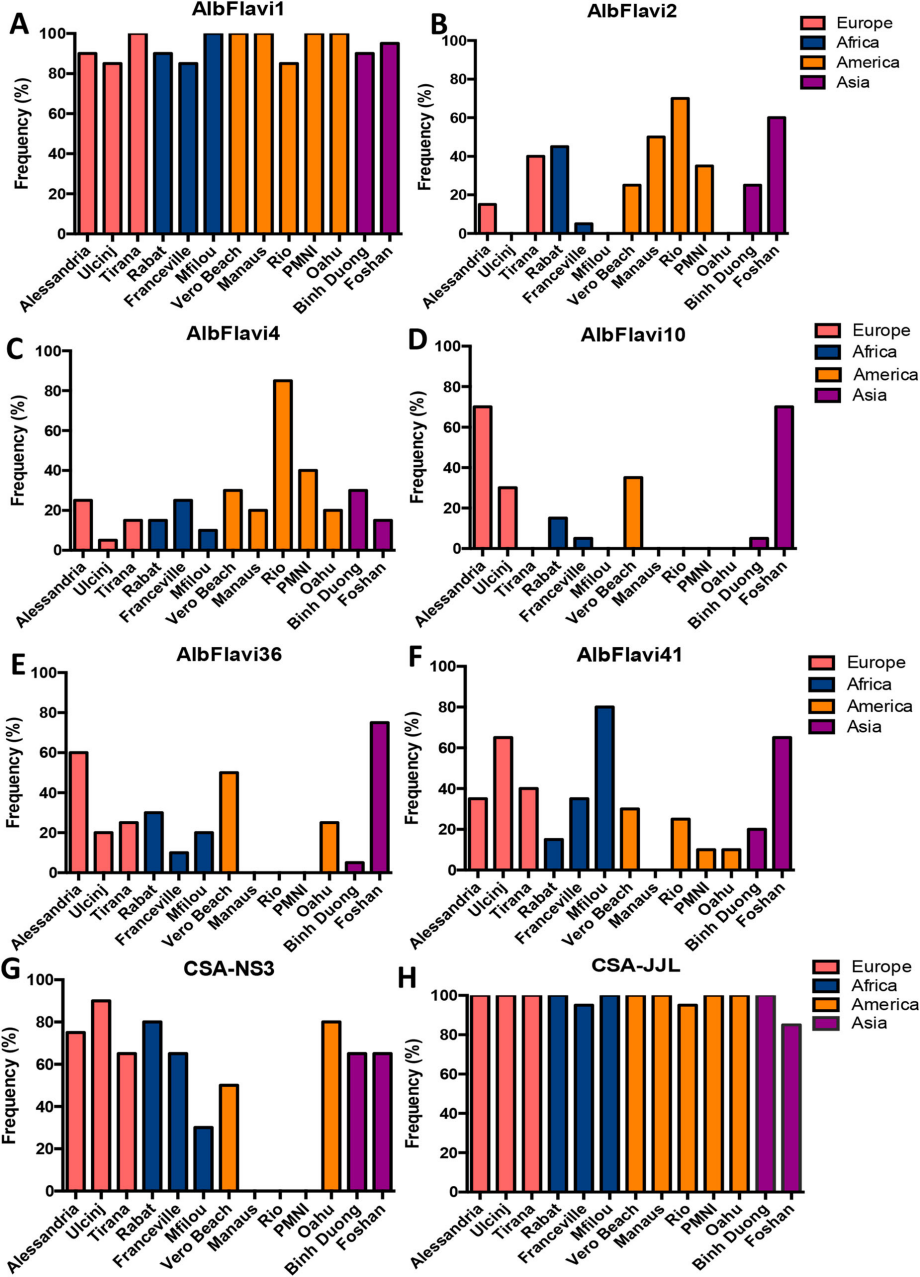
NIRVS variability among *Aedes albopictus* populations. The frequency of AlbFlavi1 (A), AlbFlavi2 (B), AlbFlavi4 (C), AlbFlavi10 (D), AlbFlavi36 (E), AlbFlavi41 (F) and CSA (G and H) was assessed for 20 individuals in each *Ae. albopictus* population (except the Rio population with 19 individuals). Populations were clustered according to their continent of origin. Oahu and Foshan correspond to laboratory colonies. The variability of CSA was assessed using two sets of primers: CSA-NS3 (G) and CSA-JJL (H).

We further chose to study NIRVS distribution in 13 *Ae. albopictus* populations selected previously for assessing genetic diversity based on epidemiological data of arboviral outbreaks and *Ae. albopictus* widespread out if its native range [17,34,35]; we analyzed 10 females and 10 males per population (Table 1). NIRVS distribution was not homogenous across populations (Figure 2, Supplementary Figure 2). AlbFlavi1 (Figure 2(A)) and CSA-JJL (Figure 2(H)) were the most widespread NIRVS, being detected in 85–100% of tested mosquitoes, respectively. On the opposite, AlbFlavi10 was the rarest NIRVS, being found in 17% of the tested mosquitoes. Despite displaying the highest presence of AlbFlavi2 and AlbFlavi4 (present in most populations), Brazilian populations showed the lowest number of NIRVS, with the absence of AlbFlavi36 and AlbFlavi10 and the lower frequency of AlbFlavi41 compared to the other *Ae. albopictus* populations (Figure 2).

Interestingly, when targeting the CSA locus, no amplification was obtained for Brazilian mosquitoes using the NS3-CSA primer set (Supplementary Table 1; Figure 2(G)), which was not consistent with the results obtained with the JJL-CSA primer set that amplified the same NIRVS but targeting a different region (Figure 2(H)). This suggests then that recombination events occurred at this genomic position for Manaus, Rio and PMNI mosquitoes.

#### Comparison between microsatellite data and NIRVS abundance profiles

**Figure 3.**
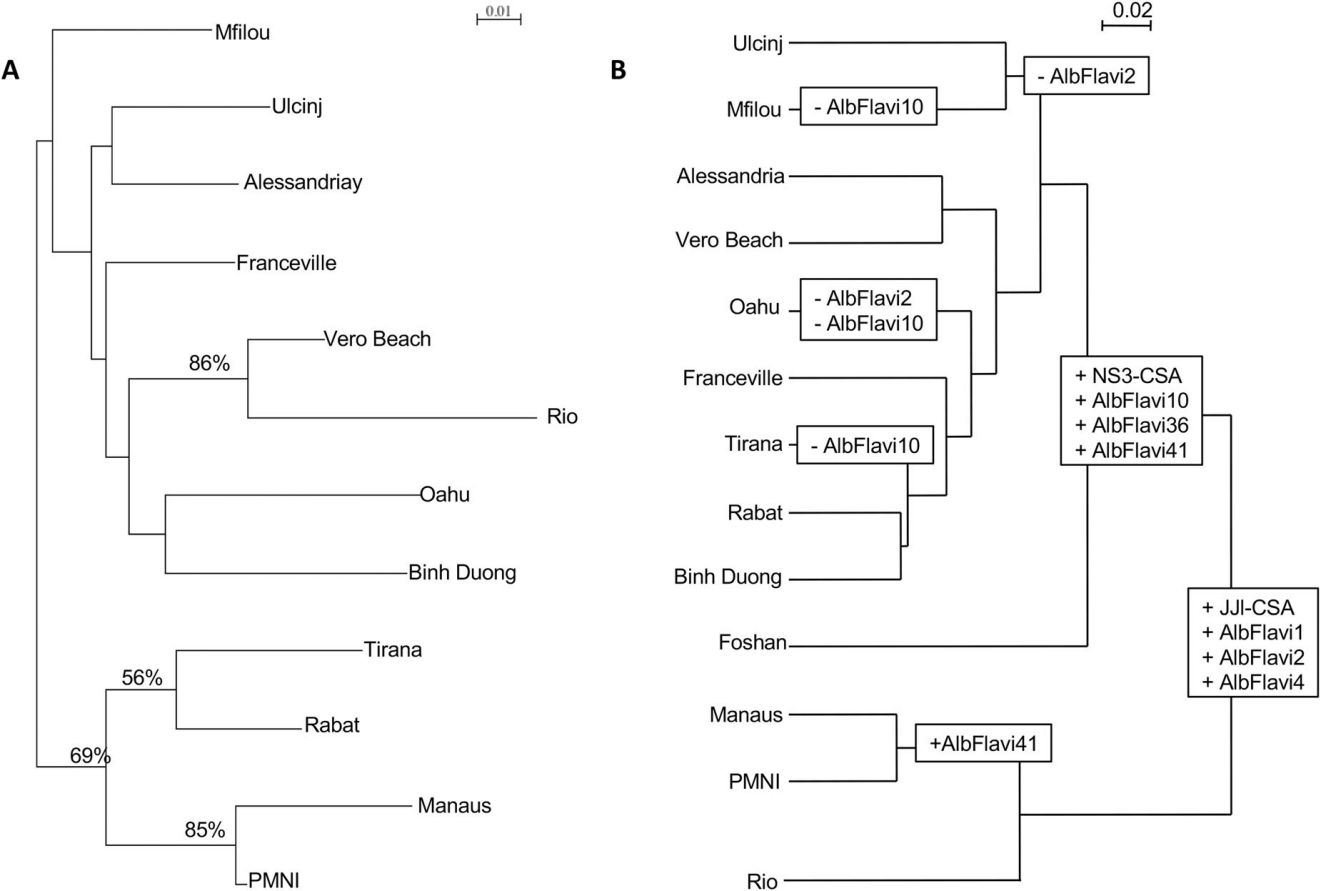
*Aedes albopictus* population clustering based on microsatellite and NIRVS loci. (A) Dendrogram of *Ae. albopictus* populations based on the analysis of 8 microsatellite loci of 12 *Aedes albopictus* populations using Cavalli-Sforza & Edwards’s genetic distance and Neighbour-Joining method. Bootstrap values were indicated when >50%. (B) Dendrogram of *Ae. albopictus* populations based on Bray Curtis distance representing dissimilarities between NIRVS composition and abundances.

To define whether NIRVS evolve under selective pressures or randomly, we compared (i) the relatedness between populations based on microsatellite polymorphism (Figure 3(A)) with (ii) the similarity between populations based on NIRVS abundance profiles (Figure 3(B)). At the population level, the Neighbour-Joining clustering analyses applied to genetic frequencies (Figures 1 and 3(A), Supplementary Figure 1(B)) mainly revealed two clusters of populations: one with Tirana, Rabat, Manaus and PMNI populations, and the second with the remaining populations (i.e. Binh Duong, Oahu, Vero Beach, Rio, Franceville, Alessandria, Ulcinj and Mfilou). Regarding NIRVS contents, two major clusters distantly related from each other were also obtained (Figure 3(B)), one including only Brazilian populations (i.e. Manaus, PMNI and Rio) sharing a low abundance of NIRVS, and the other including geographically distant populations. Therefore, populations from Vero Beach and Rio shared closely related genetic relationships and harboured clearly different abundance profiles of NIRVS (Figure 3(A,B)). Conversely, genetic distinct populations from Vero Beach, Rabat and Alessandria shared the same contents of NIRVS. In short, closely related populations can have different NIRVS contents and geographic and/or genetic distant populations can contain similar NIRVS abundance profiles. Thus, we observed significantly different relatedness between populations according to the marker used, that may indicate that random genetic drift was not the main force shaping these NIRVS distribution. In addition, no correlation was identified between the two matrices (*R*^2^ = 0.0006), showing that NIRVS composition of populations was not related to the genetic structure caused by random genetic drift (Supplementary Figure 2).

### Relationships between NIRVS landscape and vector competence (from published data)

Our results showed that NIRVS were not neutrally distributed across populations supporting the hypothesis of a biological function of NIRVS, such as antiviral functions, as it has been previously suggested. To analyze whether NIRVS contribute to the control of arboviral replication, we used logistic regression models to evaluate potential association between NIRVS distribution frequencies among populations and dissemination efficiencies (DEs) of DENV and CHIKV in corresponding or geographically close populations selected from the literature (Supplementary Table 2). On this basis, we classified our tested populations depending on their frequencies of each NIRVS using the median. Several associations between NIRVS and DENV DEs were found. Whereas high AlbFlavi2 and CSA-JJL frequencies were significantly associated with high DEs, high AlbFlavi10, AlbFlavi36, AlbFlavi41 and CSA-NS3 frequencies were however correlated to low DENV DEs (Table 4). Opposite associations to CHIKV DEs were also observed but with fewer NIRVS. Indeed, high frequencies of AlbFlavi4 were associated with high CHIKV DEs while high distribution of AlbFlavi36 and CSA-NS3 among populations was correlated to low CHIKV DEs. Together, these results indicated that the presence of NIRVS was associated with changes in DEs of arboviruses in *Ae. albopictus* at the population level.

**Table 4.** Association between NIRVS and arboviral dissemination efficiencies in *Aedes albopictus* populations (logistic regression models).

NIRVS	*N*	OR (95% CI)	*p*-value
*DENV*			
AlbFlavi1	707	0.82 (0.61–1.11)	NS
AlbFlavi2		3.07 (2.3–4.2)	***
AlbFlavi4		1.46 (1.0–2.1)	NS
AlbFlavi10		0.46 (0.3–0.6)	***
AlbFlavi36		0.46 (0.3–0.6)	***
AlbFlavi41		0.43 (0.29–0.62)	***
CSA-NS3		0.68 (0.5–0.9)	**
CSA-JJL		3.87 (1.0–14)	*
*CHIKV*			
AlbFlavi1	360	1.53 (0.9–2.6)	NS
AlbFlavi2		1.3 (0.7–2.1)	NS
AlbFlavi4		2.14 (1.2–3.6)	**
AlbFlavi10		0.60 (0.3–1.0)	NS
AlbFlavi36		0.36 (0.2–0.6)	***
AlbFlavi41		0.61 (0.4–1.0)	NS
CSA-NS3		0.56 (0.3–1.0)	*
CSA-JJL		0.68 (0.3–1.7)	NS

Notes: Dissemination efficiencies (DE) data were assessed from the same or geographically close *Ae. albopictus* populations (see Supplementary Table 2). Populations were characterized as high or low frequencies for each NIRVS by using the median and were analyzed with DE data by logistic regression models to find any association. Odds ratio (OR) >1 and <1 indicated positive and negative association respectively. 95% CI: 95% confidence intervals; N: sample size.

### Focus on AlbFlavi2 and AlbFlavi36

Besides its association to high DENV DEs, we further focused on AlbFlavi2 located in a piRNA cluster and presenting a large geographical distribution encompassing all continents. Moreover, AlbFlavi36 located in an intergenic region was not found in populations from South America and presented an opposite pattern of association to DENV DEs; it was used for comparison to AlbFlavi2. Therefore, sequence polymorphism, evolution and potential association to vector competence to arboviruses were assessed in *Ae. albopictus* at the individual level.

#### Sequence polymorphism and evolution of AlbFlavi2 and AlbFlavi36

AlbFlavi2 and AlbFlavi36 of individual mosquitoes were successfully amplified in 10 out of the 13 *Ae. albopictus* populations studied (Figure 2). Sequences of AlbFlavi2 and AlbFlavi36 amplified fragments were aligned with the closely related exogenous viral sequences: the Kamiti River virus (KRV) for AlbFlavi2 and the Aedes Flavivirus (AeFV) for AlFlavi36.

**Figure 4.**
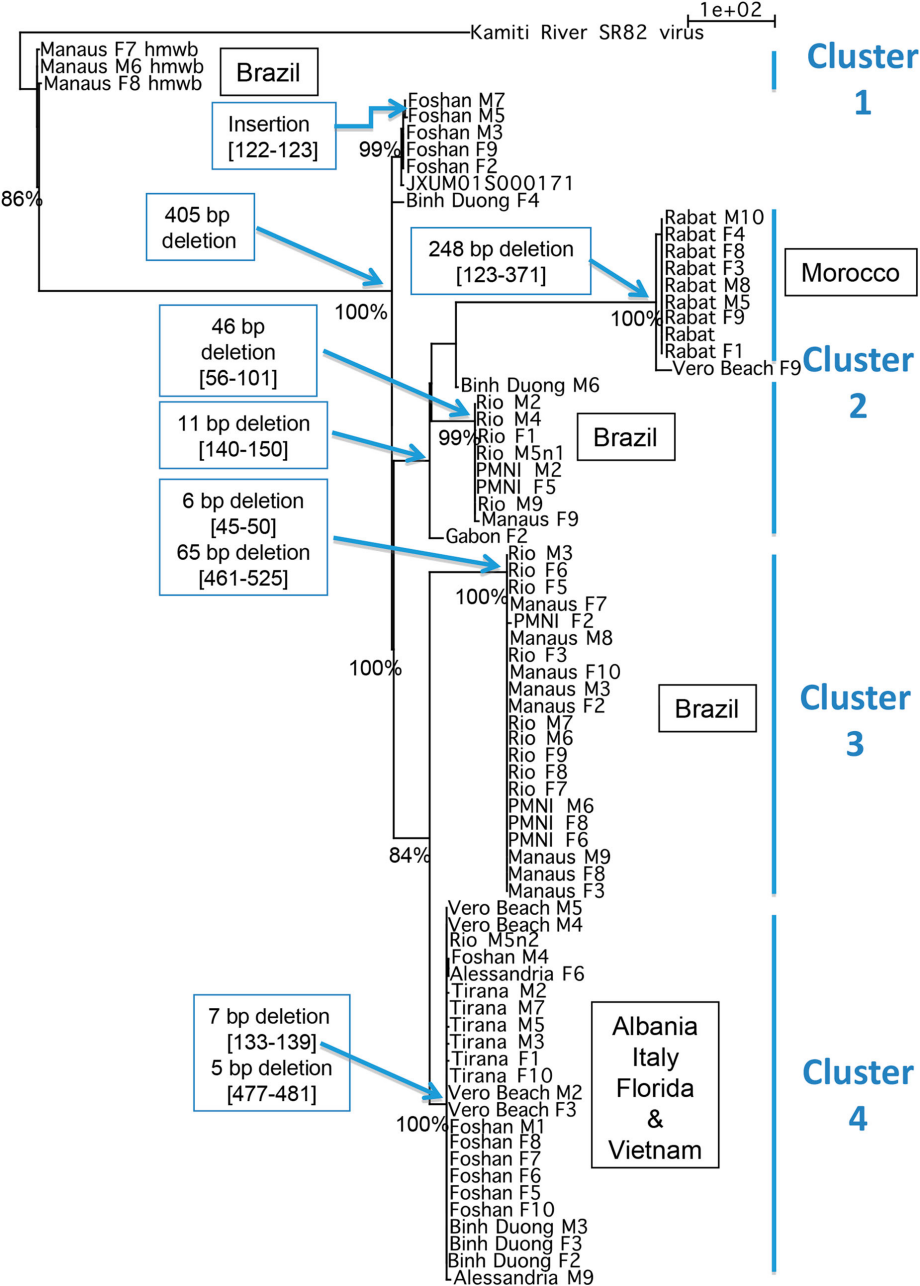
Divergence of AlbFlavi2 among *Aedes albopictus* individuals. Phylogram of AlbFlavi2 sequences based on parsimony with gaps considered as 5th nucleotides. Each node was found in 98–100% of the trees obtained through NNI rearrangements. Significant bootstrap values were indicated at nodes. hmwb: high molecular weight band Values; in brackets: alignment coordinates of deletion. The same result was obtained by parsimony without gap as 5th nucleotide, except for the sequence cluster of mosquitoes from Morocco.

The p-distance calculated between AlbFlavi2 sequences revealed a moderate diversity, with values ranging from 0% to 3.9% (data not shown). All AlbFlavi2 sequences underwent deletion events that were sometimes shared by several individuals from different populations or observed in a specific *Ae. albopictus* population (Figure 4; Supplementary AlbFlavi36).

The p-distance values calculated between AlbFlavi36 sequences were low and varied only from 0% to 0.9% (data not shown). Therefore, AlbFlavi36 sequences mapping to an intergenic region [24] appeared more similar between individuals than AlbFlavi2 sequences (which are integrated in piRNA cluster 27 [53]).

We further performed phylogenetic analysis to describe the evolutionary history of AlbFlavi2 and AlbFlavi36 (Figure 4; Supplementary Figure 4). As expected, AlbFlavi2 displayed higher divergence than AlbFlavi36. The resulted trees based on AlbFlavi2 supported four major clusters sharing sequences with the same deletion events (Figure 4). One of them, defined by a 11 bp deletion was subdivided into two subclusters of sequences sharing respectively a 248 bp and a 46 bp deletion. Overall, these clusters did not show any strict relationship with geographical origin of populations.

AlbFlavi2 from Brazilian populations (PMNI, Manaus and Rio) appeared clearly polyphyletic. Three clusters represented only sequences from Brazil: an older cluster of sequences sharing a 405 bp insertion and two other clusters of sequences sharing respectively a 46 bp and a 6–65 bp deletions. Only AlbFlavi2 from Rabat population formed a monophyletic group, a unique cluster of sequences characterized by a 248 bp deletion.

One cluster contained most of the sequences from Tirana (Albania), Vero Beach (USA), Alessandria (Italy) and Binh Duong (Vietnam). However, some sequences from Binh Duong, Vero Beach and Alessandria populations, were also isolated or found in other clusters. Moreover, half of the AlbFlavi2 sequences from Foshan formed a unique cluster whereas the other half shared the heterogeneous cluster of sequences described above.

The polymorphism of AlbFlavi36 was low in *Ae. albopictus* populations and all the sequences showed close relationships with each other, without any significant bootstrap values (Supplementary Figure 4). However, sequences from African mosquitoes (Congo, Gabon and Morocco) in branching deeply in the tree may be more ancient than sequences of Asian (Binh Duong, Foshan) and European mosquitoes.

Collectively, phylogenetic studies revealed different histories of NIRVS in mosquito populations and particularly, complex history of Albflavi2 evolving by both mutation and deletion events.

#### Association between AlbFlavi2/Albflavi36 and vector competence in Ae. albopictus at the individual level

**Figure 5.**
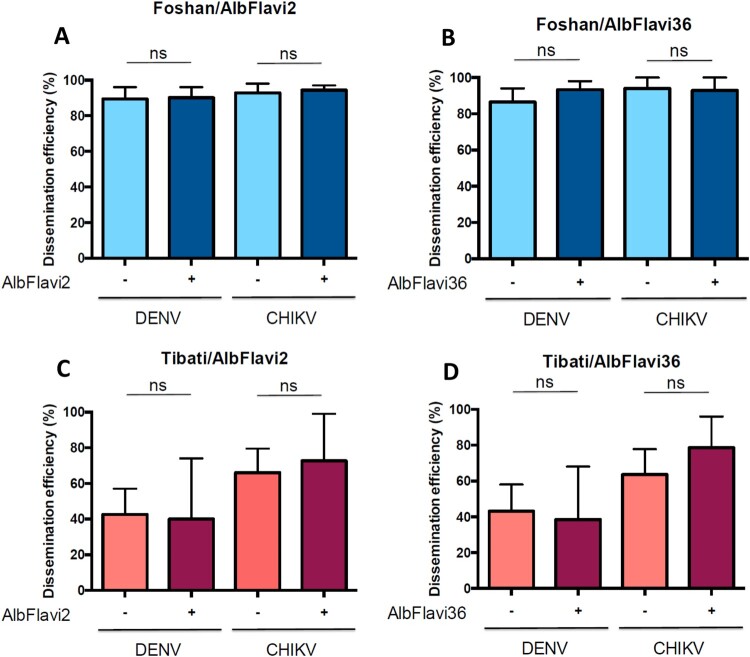
Pilot analysis showing the association between frequencies of AlbFlavi2/AlbFlavi36 and DENV/CHIKV dissemination efficiencies (DE) in *Aedes albopictus* populations. The Foshan colony and the Tibati population (Cameroon, generation F1) were used for the analysis. (A) Presence/absence of AlbFlavi2 and DEs to DENV and CHIKV obtained for the Foshan colony. (B) Presence/absence of AlbFlavi36 and DEs to DENV and CHIKV obtained for the Foshan colony. (C) Presence/absence of AlbFlavi2 and DEs to DENV and CHIKV obtained for the Tibati population. (D) Presence/absence of AlbFlavi36 and DEs to DENV and CHIKV obtained for the Tibati population. DEs were obtained for both viruses at 14 days post-infection. In total, 191 and 122 individuals were examined for presence of AlbFlavi2/AlbFlavi36 after infection DENV and CHIKV, respectively. Interactions of populations and frequencies of AlbFlavi2/AlbFlavi36 with DEs were tested using logistic regression models.

To further investigate the biological role of AlbFlavi2 and AlbFlavi36, we extended our analyses of association between the presence of these two NIRVS and viral dissemination from a population level to an individual level. To do so, mosquitoes of the Foshan colony strain and the field-collected Tibati population (Cameroon) were infected with DENV-2 and CHIKV. At 14 days post-infection, mosquito dissemination status and the presence of both AlbFlavi2 and AlbFlavi36 were determined for 313 mosquitoes (Figure 5). When comparing DENV dissemination, Foshan better disseminated than Tibati (respectively 89.8% and 42.2%; *p*-value <10^−4^; data not shown). However, the presence of AlbFlavi2 or AlbFlavi36 in mosquitoes from Foshan or Tibati was not significantly associated to DENV dissemination (*p*-values > 0.209; Figure 5(A–D)). Moreover, the same results were obtained regarding the association between AlbFlavi2/AlbFlavi36 presence and CHIKV dissemination (*p*-values > 0.3; Figure 5(A–D)). In all, using both laboratory colony and field-collected mosquitoes, no association was found between any of the two NIRVS (AlbFlavi2 and AlbFlavi36) and DENV/CHIKV dissemination.

## Discussion

The presence of sequences with similarities to Flaviviruses in the genome of *Aedes* spp. mosquitoes and their enrichment in piRNA clusters support the hypothesis that viral integrations, at least some, are not simply viral fossils, but could have a biological role [24,25,54]. Here we demonstrated that the NIRVS studied are not neutrally distributed among *Ae. albopictus* populations and all of them (except AlbFlavi1) were significantly associated to vector competence to DENV and CHIKV.

### NIRVS are not neutral markers

NIRVS are endogenous viral sequences located in protein-coding gene exons, intergenic regions, and PIWI-interacting RNA (piRNA) clusters [55]. Contrary to the other categories, piRNA genes are evolving rapidly under positive selection to generate a high diversity of piRNAs [56–63]. Approximately 30 sequences of flaviviral ORFs (primarily NS1 and NS5) were detected in the Foshan colony [24] including the early-detected Flavivirus-like sequences [21,64]. It has been suggested that the variable number and frequency of NIRVS across *Ae. albopictus* populations contribute to variations of genome size among mosquitoes [36]. Our study targeted seven NIRVS (AlbFlavi1, AlbFlavi2, AlbFlavi4, AlbFlavi10, AlbFlavi36, AlbFlavi41, CSA), for which their evolution under selection pressures or simply by genetic drift remained unknown.

Contrary to NIRVS deriving from flaviviruses in *Ae. aegypti* (unpublished data), the presence of the tested NIRVS was highly variable among worldwide populations, with the lowest number being detected in mosquitoes from Brazil. This suggests that these NIRVS have not reached fixation in any of the *Ae. albopictus* populations tested (with potential exception for AlbFlavi1 and a fragment of CSA). Interestingly, Brazilian populations (Manaus, Rio, and PMNI) displayed the lowest number of NIRVS studied with a total absence of AlbFlavi10, AlbFlavi36 and CSA-NS3. Considering the ancient origin of NIRVS, estimated between 6.5 thousands to 2.5 million years ago [65], we hypothesized that they might have been less exposed to ISFs in the past, leading to fewer NIRVS in their genomes or that populations may have acquired NIRVS in the past which have been lost over time.

We showed that the microsatellite polymorphism of populations did not match with the NIRVS abundance profiles. Therefore, because microsatellites are sequences that neutrally evolve in the genome, we speculate that the evolution of NIRVS is far from neutral and NIRVS could provide benefits to the host. NIRVS may have been produced for specific purposes in the host rather than being the consequences of random endogenization of exogenous viral fragments.

The focus on NIRVS from piRNA cluster (AlbFlavi2) and intergenic region (AlbFlavi36) revealed different sequence polymorphism and evolutionary histories despite their relatively wide distribution among *Ae. albopictus* populations. Whereas AlbFlavi36 appeared monophyletic with highly conserved sequences among populations, the phylogenetic analyses showed a particularly complex history for Albflavi2 evolving by both mutation and deletion events. We postulate that owing to its high variability, AlbFlavi2 may act similarly to TE fragments and piRNA genes, and evolve according to exposures to related exogenous virus burden.

### Association between NIRVS and vector competence to arboviruses

Similar to some endogenous retroviruses (ERVs) that have an effect on viruses from different subfamilies and genera [66], we questioned whether NIRVS deriving from ISF sequences affect the dissemination of different arboviruses and contribute to the regulation of vector competence of *Ae. albopictus* mosquitoes. Because NIRVS located in piRNA clusters, such as AlbFlavi2, produce piRNAs, are differentially distributed among populations and appeared older integrations than NIRVS located in codons [65], they were proposed to function as novel mosquito antiviral immune factors. However, contradictory results were obtained using both population- and individual-level analysis.

At the population level, many NIRVS were present in *Ae. albopictus* populations. The frequency of some of them appears correlated (positively or negatively) to DENV dissemination. Whereas some NIRVS were associated with high viral dissemination (i.e. AlbFlavi2 and CSA-JJL), others were significantly related to low viral dissemination (AlbFlavi10, AlbFlavi36, AlbFlavi41 and CSA-NS3), suggesting different functions of NIRVS deriving from ISFs. Moreover, fewer NIRVS were positively (i.e. AlbFlavi4) and negatively (i.e. AlbFlavi36 and CSA-NS3) correlated to CHIKV dissemination. These results are consistent with the contradictory results obtained in studies assessing the impact of exogenous ISFs on arbovirus fitness in mosquitoes. Indeed, whereas some ISFs have shown to repress arboviral replication [67], others have been proved to facilitate infection [68]. Further experiments should be performed to confirm these results, as only 20 mosquitoes per population were tested for NIRVS distribution.

At the level of individuals, our pilot experiment based on mosquitoes from both laboratory colony and field did not show any significant association between dissemination of DENV and CHIKV, and NIRVS, AlbFlavi2 or AlbFlavi36. while this does not seem surprising for the alphavirus CHIKV considering that NIRVS examined are homologous to flavivirus sequences, it is still questionable for DENV. Since it appears that they do not evolve neutrally and were preserved from purifying selection, NIRVS such as CSA has demonstrated to produce a transcript of 4671 nt long [21,69]; its functional role should be investigated. Lastly, other components should be considered such as the virome [70] in addition to the anatomical barriers in the mosquito such as the salivary glands for viral transmission [19].

To conclude, our results clearly show that NIRVS in *Ae. albopictus* follow processes different from that of neutral genes such as microsatellites and most NIRVS are far from reaching fixation. Flaviviral integrations are differentially distributed among *Ae. albopictus* populations and are here suggested to be associated with the vector competence to arboviruses by mechanisms that remain to be elucidated. Finally, this study opens the way to new perspectives on evolution and biological functions of NIRVS, in part on vector competence.

## Supplementary Material

Supplemental Material
