# Supplementary material for: Evolution and biological significance of flaviviral elements in the genome of the arboviral vector Aedes albopictus
Source: Emerg Microbes Infect. 2019 Aug 30;8(1):1265–79. doi: 10.1080/22221751.2019.1657785 (PMC6735342; doi:10.1080/22221751.2019.1657785)
Supplement: Supplemental Material [file TEMI_A_1657785_SM4335.docx]

**Evolution and Biological significance of Flaviviral elements in the genome of the arboviral vector *Aedes albopictus***

Vincent Houé^1,2^, Gaelle Gabiane,^1^ Catherine Dauga^3^, Marie Suez^4^, Yoann Madec^5^, Laurence Mousson^1^, Michele Marconcini^6^, Pei-Shi Yen^1^, Xavier de Lamballerie^7,8^, Mariangela Bonizzoni^6^ and Anna-Bella Failloux^1*^

**Supplementary Table 1. Details on primers used to amplify fragments of NIRVS in *Ae. albopictus* genome.**


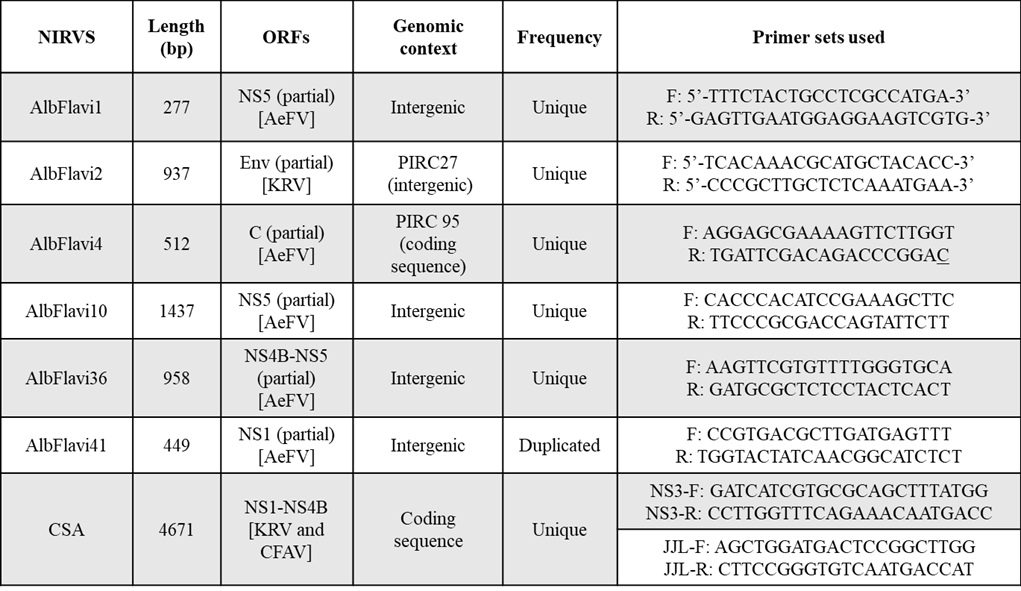


**Supplementary Table 2. Dissemination of DENV and CHIKV in *Ae. albopictus* populations.** Data are extracted from our published studies.

| Population | DENV | | |  | CHIKV | | | References |
| --- | --- | --- | --- | --- | --- | --- | --- | --- |
|  | Mean (%) | N | 95% Confidence Intervals |  | Mean (%) | N | 95% Confidence Intervals |  |
| Alessandria | 38.46 | 65 | 26.31 - 50.61 |  | 74.19 | 31 | 57.87 - 90.50 | Talbalaghi et al. (2010) ^71^ |
| Montenegro | 8.33 | 24 | 1.02 - 26.99 |  | 82.5 | 40 | 70.19 - 94.80 | Zouache et al. (2014) ^20^  Houé et al. (unpublished data) |
| Albania | 80 | 20 | 60.79 -99.20 |  | 83.33 | 18 | 58.58 - 96.42 | Houé et al. (unpublished data) |
| Gabon | 21.42 | 14 | 4.65 - 50.79 |  | 86.04 | 43 | 75.25 - 96.83 | Vazeille et al. (2008) ^72^ |
| Congo | 21.42 | 28 | 5.22 - 37.63 |  | 53.84 | 26 | 33.31 - 74.38 | Paupy et al. (2010) ^73^  Vazeille et al. (2016) ^41^ |
| Morocco | 60 | 30 | 41.39 - 78.60 |  | 53.33 | 30 | 34.38 - 72.28 | Amraoui et al. (2019) ^52^ |
| Vero Beach | 38.58 | 127 | 30.00 - 47.16 |  | 93.33 | 30 | 77.92 - 99.18 | Vega-Rua et al. (2014) ^47^  Lourenço-de-Oliveira et al. (2003) ^74^ |
| Rio | 71.14 | 149 | 63.78 - 78.50 |  | 91.30 | 23 | 71.96 - 98.92 | Lourenço-de-Oliveira et al. (2003) ^74^  Vega-Rua et al. (2014) ^47^ |
| Manaus | 50 | 30 | 31.01 - 68.98 |  | 96.66 | 30 | 82.78 - 99.91 | Houé et al. (unpublished data)  Vega-Rua et al. (2014) ^47^ |
| PMNI | 58.97 | 117 | 49.92 - 68.01 |  | 89.79 | 49 | 81.01 - 98.58 | Lourenço-de-Oliveira et al. (2003) ^74^  Vega-Rua et al. (2014) ^47^ |
| Vietnam | 48.54 | 103 | 38.72 - 58.35 |  | 82.5 | 40 | 70.19 - 94.80 | Zouache et al. (2014) ^20^  Paupy et al. (unpublished data) |

Supplementary Fig. 1. Population structure analysis of 363 *Aedes albopictus* individuals based on microsatellite markers. (A) Magnitude of ΔK as a function of K (mean over 25 replicates) calculated for the model. The modal value of ΔK was K=2, indicating that individual mosquitoes were divided into 2 clusters (B). The division of the dataset in two groups corresponds to the best assignment of individuals with Structure. Little magnitude of ΔK at K=4 suggested no subclusterization for the Cluster 1, but the Cluster 2 substructured in 3 different subclusters (C).


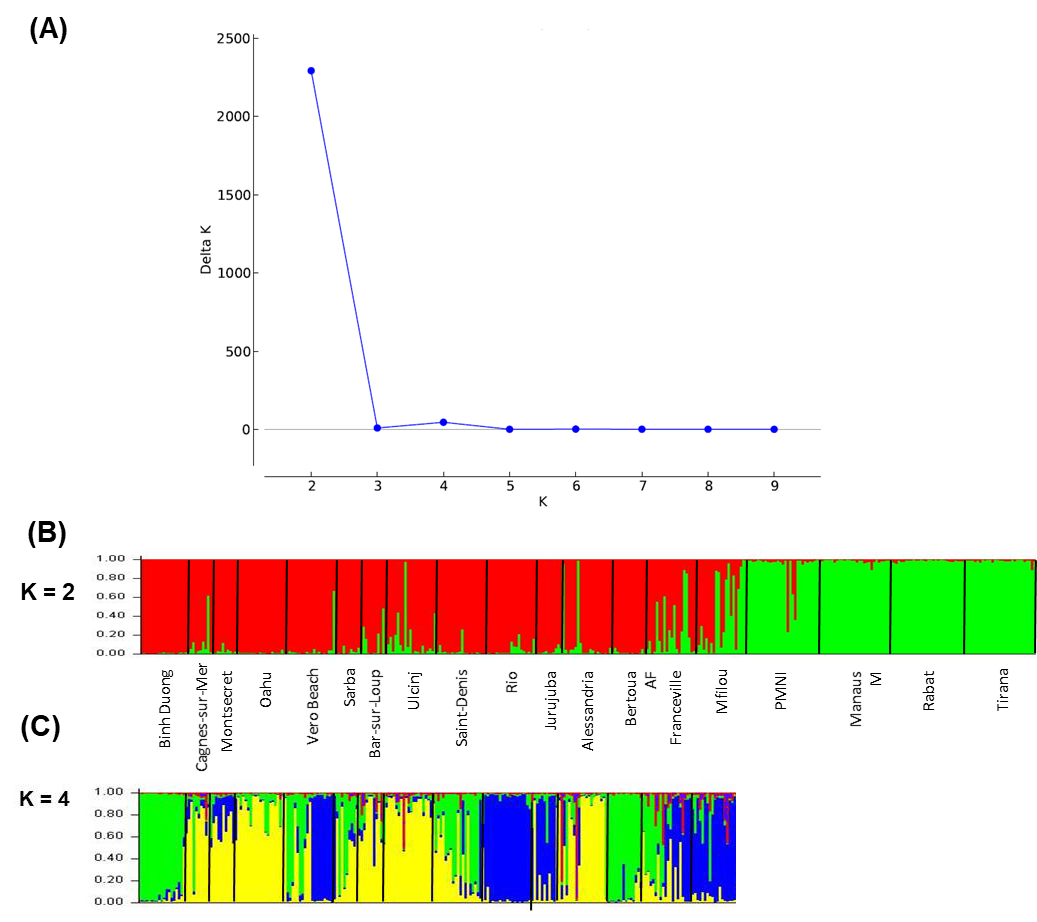


**Supplementary Fig. 2. Results of the Mantel test on** $\boldsymbol{\varphi'st}$ **matrices based on microsatellite data (x-axis) and NIRVS distribution data (y-axis).**


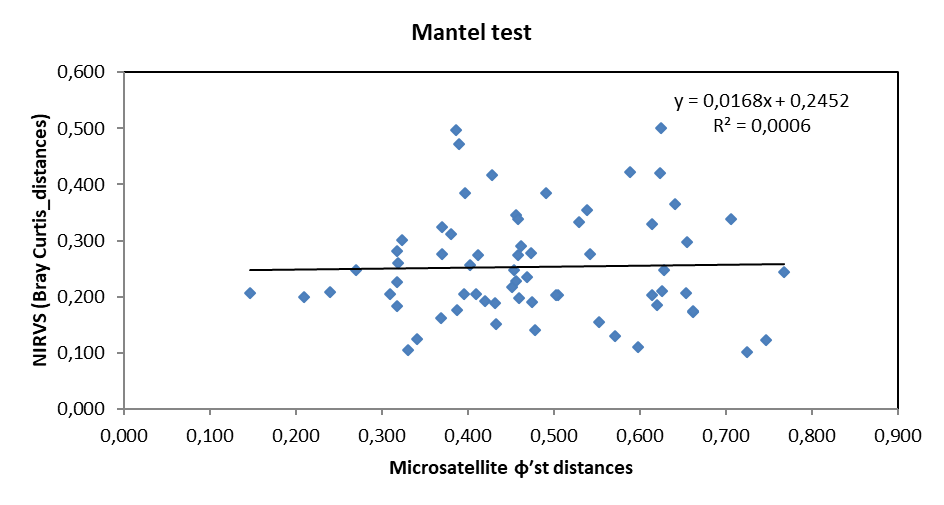


**Supplementary Fig. 3. Sequence polymorphism of AlbFlavi2 and AlbFlavi36 in *Ae. albopictus* populations**. Each population sequence (blue bars) of AlbFlavi2 and AlbFlavi36 was compared to its related viral sequence: partial envelope protein-coding sequence of Kamiti River Virus (green bar) (**A**) and partial NS4B-NS5 protein-coding sequence of Aedes Flavivirus (orange bar) (**B**) respectively. Deletions and insertions in population sequences were represented as gaps (blue lines) and orange triangles respectively, and their sizes and positions in the viral-related sequence were indicated. The number of individuals in a population displaying each indel was indicated below the event. For each population, the number of individuals positive for each NIRVS among the total individuals tested was indicated in the right column.


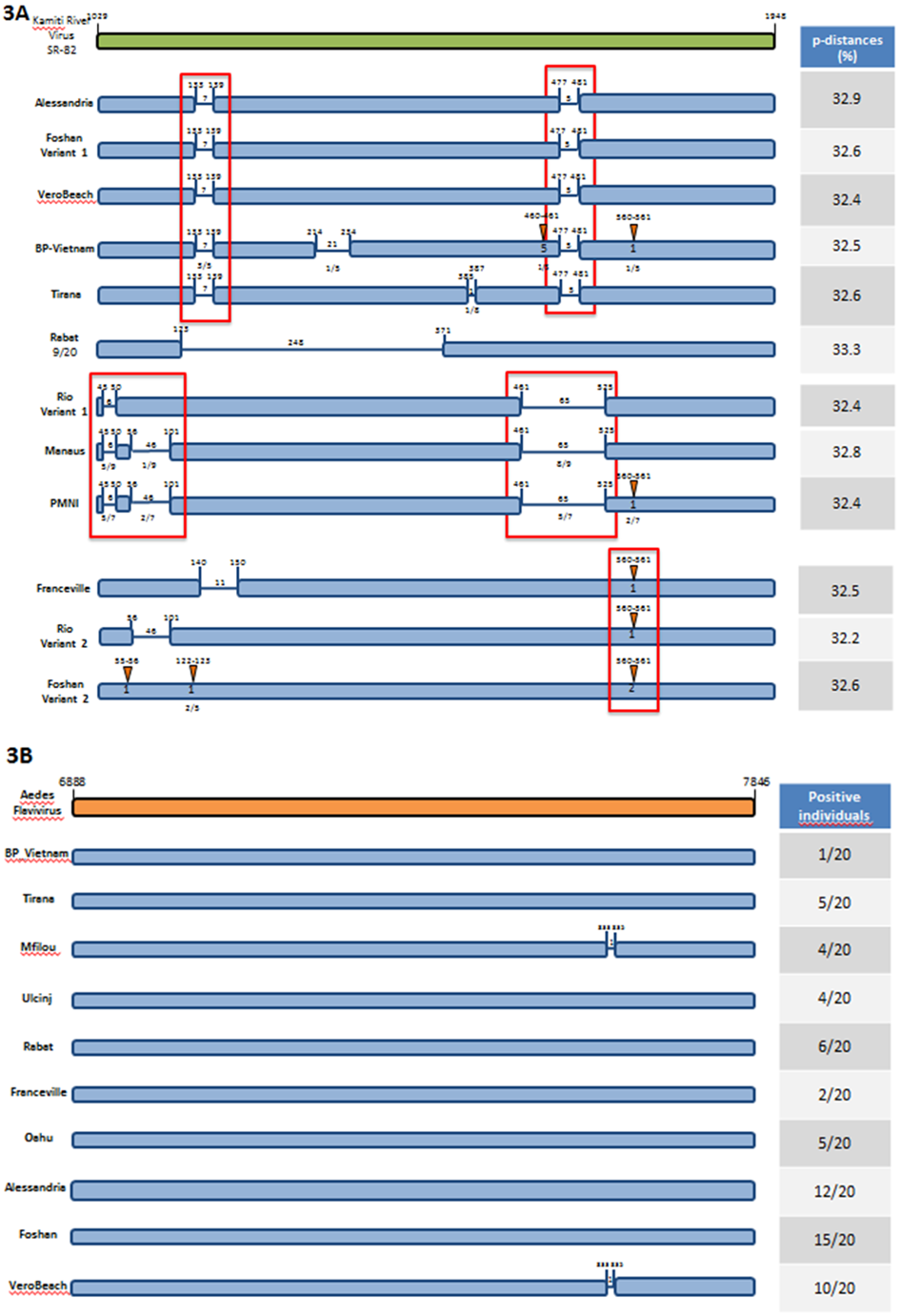


**Supplementary Fig. 4. Divergence of AlbFlavi36 among *Ae. albopictus* individuals.** AlbFlavi36 phylogram based on parsimony. Each node was found in 98 to 100% of trees obtained through NNI rearrangements.


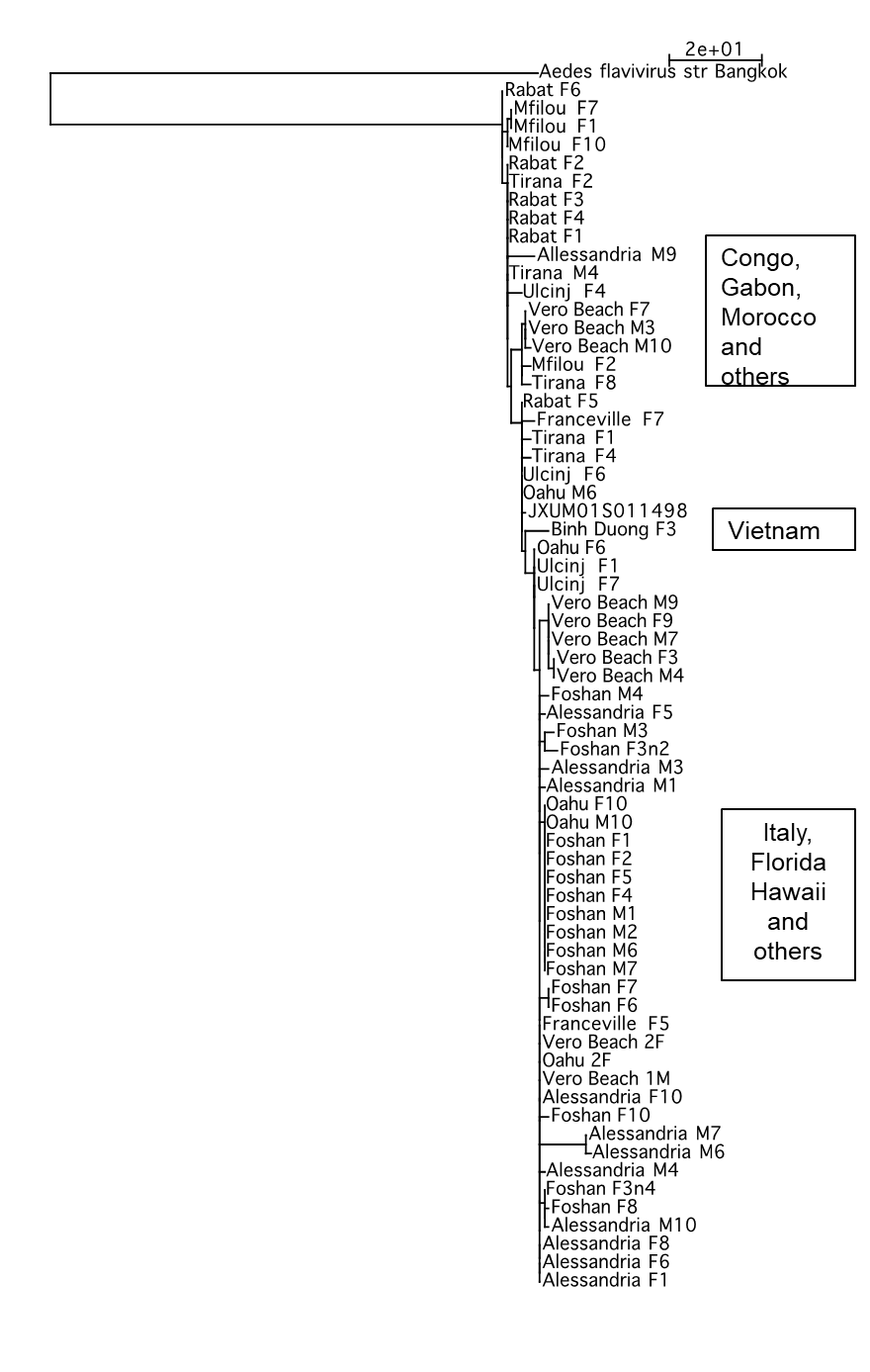


**REFERENCES**:

[71] Talbalaghi A, Moutailler S, Vazeille M, et al. Are Aedes albopictus or other mosquito species from northern

Italy competent to sustain new arboviral outbreaks? Med Vet Entomol. 2010;24:83–87. doi:10.1111/j.1365-

2915.2009.00853.x.

[72] Vazeille M, Moutailler S, Pages F, et al. Introduction of Aedes albopictus in Gabon: what consequences for dengue and chikungunya transmission? Trop Med Int Health. 2008;13:1176–1179. doi:10.1111/j.1365-3156.

2008.02123.x.

[73] Paupy C, Ollomo B, Kamgang B, et al. Comparative role of Aedes albopictus and Aedes aegypti in the emergence of dengue and chikungunya in Central Africa. Vector Borne Zoonotic Dis. 2010;10:259–266. doi:10.

1089/vbz.2009.0005.

[74] Lourenço-de-Oliveira R,VazeilleM,de FilippisAM, et al. Large genetic differentiation and low variation in vector competence for dengue and yellow fever viruses of Aedes albopictus from Brazil, the United States, and the Cayman Islands.AmJ TropMed Hyg. 2003;69:105–114.
